# Considering Sex/Gender-Based Violence as a Form of Hate: The Invisibility of Sex and Gender

**DOI:** 10.1177/15248380241311873

**Published:** 2025-01-14

**Authors:** Myrna Dawson

**Affiliations:** 1 University of Guelph, Guelph, ON, Canada

**Keywords:** community violence, domestic violence, hate crimes, sexual assault, domestic violence, homicide

## Abstract

Globally, there is no shortage of examples demonstrating lethal and non-lethal violence motivated, at least in part, by a hatred of women and girls because of their sex or gender. Such violence is not a new phenomenon. Despite this, there remains little consideration of sex/gender-based violence (S/GBV) motivated by hatred in the hate/bias crime literature, including a recent comprehensive review published in this journal. Drawing from a comprehensive scoping review of international literature, this article discusses why this might be the case, identifying both the benefits and challenges of treating sex/gender-motivated violence as a form of hate. The review examined primarily legal- and case-based analyses, grey literature, and some empirically based research articles, both qualitative and quantitative, the latter of which largely had only a peripheral focus on the question posed—the consideration or recognition of sex/gender-motivated hate that leads to violence. Themes surrounding benefits and challenges of doing so were identified. Among the findings was that, while there are valid arguments for and against the inclusion of, or emphasis on, S/GBV as a form of hate, what is largely absent from the body of literature is systematic, empirically based evidence examining the validity of the arguments identified, particularly in recent years. The article concludes by highlighting four broad research and policy priorities which can further (or arguably begin) the conversation about the role of hate in S/GBV.

## Key Findings of the Review

• Despite the recognized increase in hate crimes throughout the COVID-19 pandemic and the recognition of such, sex/gender-based violence (S/GBV) motivated by hate remains invisible, especially male violence against women and girls (MVAWG).
• Recognizing the role of multiple, and overlapping, identities and oppressions highlighted by intersectional theorists, sex or gender has seldom been prioritized, or considered equal to, race or religion for example, when discussing intersecting motivations for hate.
• A comprehensive review of the literature finds both benefits and challenges in considering S/GBV, and particularly MVAWG, as a form of hate.
• Empirical research examining S/GBV as a form of hate is largely non-existent.
• There is a seemingly entrenched historical and contemporary reluctance to recognize sex or gender as necessary protected characteristics or identifiable groups in hate crime frameworks.
• This situation stems from common beliefs about MVAWG, and specifically its normalization within a patriarchal society, including law and academia.

*Implications of the Review for Practice, Policy, and Research*

• Countries need to enhance monitoring of S/GBV as a form of hate, including data on reporting, recording, prosecuting, and sentencing with a particular emphasis on sex or gender, while recognizing the intersectional experiences of victims of hate.
• In data collection on S/GBV as a form of hate, it is important not to conflate, sex, gender, sexual orientation, and gender identity or expression. Nuanced and effective interventions require the ability to identify similarities and differences based on the protected characteristic or identifiable groups that can inform prevention.
• Given its relative invisibility as a hate crime, it will be important to examine what are currently the common understandings of S/GBV as a form of hate by legal actors, those who work with victims of S/GBV, the victims themselves, and the general public.
• It is necessary to improve existing legislation and related policies and practices when considering S/GBV as a form of hate, and specifically clear criteria for identifying when it occurs.
• To improve responsiveness to S/GBV as a form of hate, consistent and quality training for legal actors is necessary, including how to identify when S/GBV is a form of hate (hence, the need for criteria as noted above).
• More often considering S/GBV as a form of hate, when appropriate, can help reformulate perceptions of these crimes and shift the individual and cultural focus from the victim’s behavior to that of the perpetrators’ motivations, including hate/bias.

## Introduction

In recent years, there have been multiple high-profile killings of exclusively or predominantly women which clearly demonstrate, at least in part, hatred motivated by sex or gender (Marganski, 2019; Scaptura & Hayes, 2023). Some of these killings have been linked to misogynist men’s movements and/or terrorism, whereas others reflect misogynist beliefs of individual men, supported by patriarchal social structures and processes (Dawson, 2022). For example, a male adult in Canada was convicted and sentenced for killing eight women and two men and injuring 16 others after intentionally driving a van down a busy city street in 2018. He admitted that he drew his inspiration from the incel (involuntary celibate) online subculture of men united by sexual frustration and hatred of women. In 2020, another male youth attacked two women, killing one of them, and was sentenced to life in prison. The court found that he had committed terrorist activity motivated by incel ideology.

Men’s hatred of women is not limited to online or extremist subcultures, however (Bates, 2020). And, while recent cases seem to proliferate globally, such violence is not a new phenomenon. Thirty-five years ago, Canada was the location of what is commonly referred to as the “Montreal Massacre,” described internationally as an iconic example of a crime motivated by hatred on the basis of sex (Lawrence, 1999a, p. 25). In this instance, a man killed 14 women in a crowded university classroom after sending the men out into the corridor. He left behind a three-page statement in which he blamed feminists for spoiling his life thereby shooting his victims solely because they were women.

It is perplexing, then, that a recent comprehensive review of bias-motivated victimization and offending from different scientific disciplines excluded studies that analyzed gender-based violence (GBV), intimate partner violence, and domestic violence (Días-Faes & Pereda, 2022). As documented by a voluminous body of literature over past decades, women and girls bear the largest burden of these forms of violence, primarily perpetrated by men as well as sexual violence, also seemingly absent from the review article. These exclusions starkly highlight that there remains little consensus about, or even consideration of, these forms of violence as hatred or hate motivated, not just in the above article, but in the larger field. Like the review article, sex or gender is often invisible in historical and contemporary literature on hate-motivated victimization and offending (Dawson, 2022). Like the broader literature, however, the review article does consider, albeit briefly, lesbian, gay, bisexual, and transgender (LGBT) communities.

The current article asks why women and girls, historically and today, are consistently overlooked in the context of hate crime paradigms or frameworks, despite theoretically being protected in some countries’ legislative frameworks? In doing so, the article considers why male violence against women and girls (MVAWG), which may often be motivated by hatred or bias based on sex or gender, is not more frequently the focus of hate crime literature and related legal responses. This includes the broader category of violence now more often referred to as GBV which typically encompasses intimate partner violence, domestic violence, and sexual violence from which women and girls bear the largest burden. Even if one’s focus is limited to specific types of more commonly recognized forms of bias or hate, the growing awareness of the role played by intersectionality begs the question as to why race/ethnicity or religion, for instance, is not considered in combination with sex or gender (or other identities). It is well documented that varying combinations of social identities have significant impacts on the reporting, experiences of, and interventions in hate crime victimization and offending (Erentzen & Schuller, 2020; Pezzella et al., 2019).

This article proceeds in three stages. First, a brief global review is provided as to where hate or bias motivated by sex or gender is considered in legislation. Next, drawing from an international scoping review, the core focus of the article examines the various reasons found for and against considering violence motivated by sex or gender as hate crime, using rape/sexual assault and femicide as illustrative examples where appropriate. Women are much more likely to be victims of rape (or sexual assault in some jurisdictions) *because* of their sex or gender and perpetrators are primarily men (Walters & Tumath, 2014). Similarly, femicide is defined as the sex- or gender-related killing of women and girls *because* of their sex or gender, also perpetrated primarily by men (Dawson & Mobayed Vega, 2023). As a result, these two types of violence provide the clearest illustrative examples of sex- or gender-based violence which may be hate motivated, as Walters & Tumath (2014) argue:

“As with other forms of prejudice, misogyny can be described as a type of hatred of women, or within the legal lexicon, gender-based ‘hostility’.” Men are taught to harbor gender animus as a result of their socialization process, and in the context of structural power relations between men and women, learn to see – and denigrate – women as inherently inferior” (p. 572).

This section expands on issues highlighted in the introduction, including how violence motivated by hatred on the basis of sex or gender shares similarities to more commonly recognized targets of hate (e.g., religion, race). The article concludes by briefly highlighting research and policy priorities which can move this conversation forward, including how future research can help shift the individual and cultural focus of hate crimes from the victims’ identity and/or their culture to perpetrators’ perception of their victims’ identities and/or cultures, leading to their subsequent hate-motivated actions. For women and girls targeted because of their sex or gender, this shift can refocus attention from victim blaming narratives to perpetrator motivations, including the role played by misogyny.

It is important to briefly highlight the context within which this article was written, its terminology and scope. First, while they are distinct terms, sex and gender are used interchangeably in this article to reflect the fact that they are now conflated in most hate crime legislation as well as in academic studies (Haynes & Schweppe, 2020). Moreover, when included in hate legislation, what term is used varies across jurisdictions with some countries identifying “sex” (e.g., Canada) and others using “gender” (e.g., United States). Terms also differ with hate, bias, animus, hostility, and so on used to capture such motivations, depending on the jurisdiction. Therefore, this article will use what is appropriate for each jurisdiction; however, for general reference, “sex” and “hate” will be used given it is the terminology most often adopted in Canada, the jurisdiction in which the author resides. Consequently, sex/gender-based violence (S/GBV) will be used to capture the various forms of violence disproportionately impacting women and girls, typically absent from hate crime literature, including intimate partner and domestic violence, rape/sexual assault, and femicide. At times, sex and gender (or women and girls) and S/GBV will also be used interchangeably as it is in the literature.

Second, some jurisdictions do have the capacity to consider sex or gender in their hate crime frameworks because this characteristic is already protected or listed as an identifiable group. However, sex is rarely the focus of law in practice. Therefore, the article will often refer to the *inclusion of*, or *emphasis on*, this characteristic as a form of hate to capture that, even when included in “law on the books,” it largely remains invisible in “law in practice.” As will be shown, there is a well-documented historical and contemporary resistance to including sex as a protected characteristic or identifiable group in many countries or world regions, even when other characteristics such as sexual orientation or gender identity or expression are included. In fact, research has shown that there has been more acceptance over time, especially by government bodies, of sexual orientation and, more recently, gender identity or expression as motivations for hate (Haynes & Schweppe, 2020). For this reason, this article focuses on sex or gender—and hate motivated by one’s sex or gender—which ultimately leads to a disproportionate amount of S/GBV against women and girls in its various forms.

Third, literature on hate and hate crime is voluminous and diverse, crossing various disciplines and spanning many decades, with varying definitions of hate, including hate speech. This article focuses specifically on sex/gender-motivated hate that leads to violence to keep the scope of the article manageable. It is acknowledged, however, that hate speech can often be violent and experienced as such by those targeted and requires a separate and comprehensive examination.

Finally, this article draws from the definition of hate (or hate crime) identified in the review article which prompted this discussion. Summarizing prior literature, Días-Faes and Pereda (2022) stated that “the concept of bias/hate crime is a social construct that has been used variably to describe *antisocial and criminal behavior motivated by hate, hostility, or prejudice based on the victim’s vulnerability or the offender’s choice* (italics added)” (p. 938). The authors acknowledge, however, that there is a “certain conceptual disparity in the legal definitions, and the gaps that remain, which portrays an ‘ideal victim’” (pp. 938–939). Put another way, there exists a hierarchy of victim status which may obscure some types of bias-motivated incidents, such as those motivated by sex or gender. To respond to this situation, the authors use “bias-motivated violence as a *more inclusive term* [italics added] in which different types of targeted violence converge addressing the intersection process of the multiple factors involved and its mechanisms, as well as the effects of this type of violence on victims and their groups or communities” (p. 939). There is some ambiguity as to how this definition is more inclusive, however, since major forms of violence which disproportionately impact women and girls remain excluded. This is the crux of the matter focused upon in the current article.

## Global Considerations of Sex or Gender as Motivations for Hate

Hate crime frameworks or legislation vary significantly globally as well as within countries (e.g., United States) or world regions (e.g., Europe) with most data coming from high-income countries. Differences range from whether such legislation exists at all to what groups are protected, related offenses, and/or sentencing options (Perry, 2016; van der Aa et al., 2021). In Europe, race, national/ethnic origin, and religion are more commonly protected followed by sexual orientation and color (van der Aa et al., 2021). Less popular grounds for protection are sex or gender, gender identity, mental/physical disability, age, and language. However, van der Aa et al. (2021) noted that gender identity is becoming more important and “could be considered an emerging trend in Europe,” whereas sex remains a less popular ground (p. 175). According to the Organization for Security and Co-operation in Europe’s (OSCE) Office for Democratic Institutions and Human Rights (ODIHR), only 10 countries report sex/gender-based hate crime, including Australia, Belgium, Canada, Croatia, Georgia, Germany, Malta, Romania, Spain, and the United States.

In England and Wales, five characteristics are recognized: race, religion, sexual orientation, disability, and transgender identity. In a recent consultation by the U.K. Law Commission (2021), three options for reform were considered with respect to sex or gender: (a) full recognition as other characteristics; (b) partial recognition with certain offences and contexts excluded; and (c) no recognition of sex or gender in either aggravated offences or enhanced sentencing (p. 188). The final recommendation of the commission was that sex and gender should not be added.

In Canada, sex, sexual orientation, and gender identity or expression are designated identifiable groups in various legislation, such as the *Canadian Charter of Rights and Freedoms* (e.g., Section 15(1)), the *Canadian Human Rights Act* (CHRA) and the *Criminal Code of Canada.* Finally, in the United States, at the federal level, sexual orientation, gender, and gender identity are included among the protected groups; however, different bias motivations can be recognized at the state level with most states having protections for race, ethnicity, and religion and about two-thirds for gender (United States Department of Justice Hate Crimes, Laws and Policies (https://www.justice.gov/hatecrimes/laws-and-policies).

In summary, sex/gender remain unpopular grounds for hate-motivated crimes despite commonalities with other targets of hate in terms of general origins (e.g., discrimination based on sexism compared to racism) and impacts (e.g., instilling fear in a community or group) which will be discussed in detail throughout this article. Two similarities are briefly introduced here to show the parallels of sex-motivated hate with more commonly recognized hate motivations such as race or religion. First, because hate crimes are seen to target both individuals as well as their entire community, such acts have been referred to as “message crimes” that demonstrate to those targeted that they are not wanted or valued (Erentzen & Schuller 2020; Perry & Alvi 2012). This aptly describes the message for many victims of S/GBV, who are primarily women and girls. For example, Abir and Zrizi (2023) argue that “femicide is mainly related to the ubiquity of the male culture of entitlement” and is “linked to men’s tendency to consider women as mere ‘parasites’ that can be easily destroyed the moment they start to impede the systematic mechanism of the patriarchal order” (p. 37). As such, they argue that the act of femicide sends various key messages to all women, including that women are not men’s equals, women are second-class citizens, and women are the weaker sex, who have no real ability to challenge their historical and contemporary subordination. In reviewing the literature on fear of crime, Henson and Reyns (2015) argued that sex or gender may be “quite possibly the strongest and most widely accepted predictor of the fear of crime” (p. 95). Simply put, the documented incidence and prevalence of MVAWG is a daily message to all women that they must “stay in their place” (Pickup et al., 2001, p. 20).

Second, S/GBV often includes repeated and/or excessive violence, leading to significant psychological trauma which is further exacerbated by a common reluctance or failure to report their victimization (see Duggan, 2021, p.106). These forms of violence are so repeated and chronic that it has been described as “sexual terrorism” (Sheffield, 1987) and, more recently, “domestic terrorism” (Pain, 2014). Focusing on rape as an example, Abir and Zrizi (2023) argued that women often lose their ability to contribute to the development of societies due to the physical and psychological disabilities which evolve from their rape, including the ability to work or take care of their children. Even if women and girls have not been victims of rape, they are reminded daily, when hearing of other women’s victimizations, that they have little or no control over what happens to their bodies. “The fear of being raped haunts all women. Even those who have not experienced it live under the constant threat of being targeted by human predators” (Abir & Zrizi, 2023, p. 33). While such fear exists for women in most countries, it can be further exacerbated in some regions of the world for some groups of women, in particular. For example, Gqola (2015) introduced the concept of the “female fear factory” in relation to rape in the South African context, building further on this concept in a 2021 monograph of the same name. In her more recent writings, Gqola explained that “fear is produced in those rendered female in society like a product, and the process of that production is a factory” which is “necessary for patriarchal control” (as cited in Nkealah, 2023). S/GBV is one tool that keeps the female fear factory operating effectively.

In summary, hate crimes often involve victims with an immutable characteristic (e.g., sex), the interchangeability of victims (e.g., any woman will often do), and communal fear within the targeted group (e.g., evident in how women and girls structure their everyday activities and routines, if they can, to try to avoid risk of victimization; Carney, 2001). As such, sex-motivated hate that leads to violence is arguably similar to that experienced by other targets of hate. But hate is not distributed evenly across women and girls, similar to other hate groups. Some women and girls may be at greater risk of hate-motivated violence because they share one or more characteristics with various other common targets of hate across sociodemographics (e.g., Black women, Indigenous women, older women), sexual minorities (e.g., lesbian women, transwomen), professional groups (e.g., politicians, journalists, human rights’ defenders), and/or other characteristics (e.g., women with disabilities, immigrant, or refugee women). However, society’s capacity to document the incidence and prevalence of sex-motivated hate that leads to violence, including how it intersects with other social identities, is hampered by the under-reporting of hate crimes, especially those motivated by sex and gender, discussed next.

### Underreporting Sex/Gender as Hate and Its Intersection With Other Hate Motivations

Variations across hate crime legislation often arise, at least in part, from politically motivated decisions coupled with a lack of specific criteria to define hate crimes, resulting in sex or gender being less frequently the focus or, when included, most often considered a second- (or, arguably, third-) tier group (Jenness, 2002/2003). Similarly, the recording, prosecuting, and/or sentencing of hate crime are politicized processes with significant discretion available to decision-makers, if the hate crimes are reported at all. Hate crimes overall are significantly underreported (Erentzen & Schuller, 2020; Pezzella et al., 2019), but the situation appears to be exacerbated for hate motivated by sex or gender in jurisdictions which recognize this characteristic as a motivation for hate. As an illustrative example, police-reported data from Statistics Canada show that sex has never comprised more than 3% (and usually 2% or lower) of all reported hate crimes despite self-report data showing that 22% believe their hate crime victimization was at least partially motivated by their sex with respondents being primarily women (88%). The underreporting of sex as a motivation for hate is not reflective of patterns for other more commonly recognized hate motivations, however. For example, race as a motivation for hate was similar for police-reported (50%) and self-reported (49%) hate crime, and religion had higher police recorded incidents (25%) compared to self-reported (10%) data.

This situation is compounded by the hidden diversity in each category of hate-motivated violence, whether it is sex, sexual orientation, race, or religion. Recognizing the role of multiple, and overlapping, identities and oppressions highlighted by intersectional theorists (Crenshaw, 1989, 1991; Hill Collins & Bilge, 2016), forms of hate-motivated violence can be exacerbated for some groups specifically, including some groups of women and girls. However, police-reported and self-report data do not adequately allow for the documentation of the intersectionality of hate crime experiences. Police-reported hate crime typically captures a single motivation for hate-based offences, while self-report data often allows respondents to select as many motivations as applicable, but combinations are seldom examined. For example, as demonstrated by one Canadian study (Erentzen & Schuller, 2020), almost 60% of respondents selected two or more identities (49%) and, when only one self-reported motivation was selected, after race (70%), sex was the next most common motivation (12%). In addition, research by Perry (2014) examined hate against Muslim women, underscoring the intersectionality of religion, race, and gender, suggesting that women may also be more vulnerable to complex patterns of hate/bias-motivated violence (see also Mason-Bish & Zempi, 2019). Supporting this contention, recent police statistics in Canada showed hate crimes against Indigenous and Muslim populations were more likely to involve female victims (Wang & Moreau, 2022, p. 22). In addition, hate-motivated violence against Indigenous, Black, Asian, and other racialized women and girls has been well documented, but seldom linked to sexism or sex-motivated hate (Perry, 2014). Similarly, with the documented rise in alt-right and White supremist movements, acts of hate by these groups, including violence, are most often portrayed as racist rather than sexist or misogynist despite existing and documented interconnections across these types of ideological hate (McCulloch & Maher, 2022; Scaptura & Hayes, 2023).

Violence against lesbians may also be motivated by homophobia or misogyny. Duggan (2021) discussed how lesbian women’s intersectional experiences may remain invisible because their gendered and/or sexualized differences are not dominant in either domain. In another study of women with intellectual disabilities, McCarthy (2017) found that, for these women, disability hate crime, mate crime, and domestic violence all merged because gender, disability, and relationship status with the offender all contributed to the violence they experienced. As a result, there is clearly a need for an intersectional lens when understanding hate crime (Chakraborti & Garland, 2012 for full discussion), but research must also consider what motivations the victims themselves feel were present or most dominant. Currently, however, sex or gender is often ignored or marginalized when other more readily understood—and accepted—categories like race and religion are present.

## Methods

As documented above, sex or gender, historically and today, remain largely invisible in hate crime literature. The current article asks why this is the case; that is, why are women and girls often overlooked in this area, including the crimes in which they are disproportionately represented as victims (e.g., intimate partner violence, sexual violence, domestic violence). Drawing from a comprehensive scoping review of the international literature, the next section discusses why this might be the case, identifying both the benefits and challenges of treating sex-motivated violence as a form of hate. The review was conducted by the author as part of a larger initiative by the British Columbia’s Human Rights Commissioner’s inquiry into hate in the COVID-19 pandemic (BCHRC, 2023). During this period, as witnessed globally, the BCHRC noted that GBV had increased dramatically. As a result, they included a consideration of GBV as a form of hate and hate crime which is the first time an inquiry has included such a focus in Canada and believed to be the first time globally.

Scoping reviews are a relatively new approach to synthesizing evidence and, as such, there is little guidance about when or for what purpose they should be used, compared to systematic reviews (Levac et al. 2010; Munn et al. 2018). It has been argued that scoping reviews are largely used by researchers instead of systemic reviews when “the purpose of the review is to identify knowledge gaps, scope a body of literature, clarify concepts or to investigate research conduct . . . and they may also be helpful precursors to systematic reviews” (Munn et al., 2018, p. 1). Like systematic reviews, the validity of scoping reviews is dependent on rigorous and transparent methods with an emphasis on peer-reviewed search strategy (Levac et al. 2010). Given the expected paucity of literature, the eligibility criteria used for this review were broad, with some deviation from the peer-review emphasis as discussed below:

(1) The review included a focus on “sex” or “gender” in relation to hate crime, including all associated terms with search term combinations including sex or gender with hate, bias, hostility, animus, etc.).
(2) Any year of publication was included.
(3) Publications included peer-reviewed journals and empirical analyses as well as books on hate crime, theoretically focused articles, as well as legal- and case-based analyses to provide further, and important, context for addressing the question.
(4) Articles, texts, or analyses written in English were included.

Using the above criteria, a comprehensive search was conducted in interdisciplinary research databases, including Scopus, Google Scholar, Web of Science, Google to capture peer-reviewed articles, and gray literature. The latter included any relevant government or non-government organization (NGO) reports, professional association reports, and theses/dissertations. All referenced articles were reviewed by the author and thematic elements were identified that highlighted arguments outlining the benefits and challenges of (or the reasons for or against) considering sex or gender as potentially hate-motivated crimes, including GBV in its various forms. Given the breadth of products reviewed, some of which only had short but relevant sections on sex or gender as motivations for hate, the number of articles reviewed for this article were only roughly categorized and counted. They included nine books, 18 peer-reviewed articles (qualitative and quantitative), 13 government and non-government organizational reports, and 28 legal or case-based articles and analyses.

## The Benefits and Challenges of Treating S/GBV as Hate

While not exhaustive, three main benefits of including sex or gender as motivations for hate and/or treating S/GBV as hate were identified in the literature: (a) the symbolic impact; (b) increased public education and awareness; and (c) the emphasis on individual and community impacts. Given the historical resistance to including sex, gender, or S/GBV in hate legislation and accompanying frameworks, it is not surprising that a greater number of challenges were identified in the literature, including: (a) symbolism is weak without enforcement: (b) criminal legal systems would be overburdened; (c) resource intensiveness of emphasizing sex-motivated violence as hate; (d) challenge of training legal actors; (e) related offenses already addressed in existing legislation; (f) these forms of violence are different from other hate crimes; and (g) an emphasis on penalties rather than alternative, non-carceral approaches. In the discussion below, the inclusion of sex or gender as hate motivations is used interchangeably with the inclusion of sex/gender-based hate-motivated violence given that the bulk of the literature integrates, and often conflates for good reason, the two elements in their discussions.

### Benefits

#### The Symbolic Impact of Including S/GBV as Hate

A common argument for the inclusion of, or emphasis on, sex or gender as a form of hate motivation within the hate crime framework is its symbolic power and impact even if the actual practice does not occur as frequently as warranted (Gusfield, 1967; Gerstenfeld, 2004; Jenness & Grattat, 2005; Lawrence, 1999b). It is argued that hate crime legislation sends the message to the public that such crimes are unacceptable; that is, the individual acts and the biases or prejudices that motivate such acts, including S/GBV, against specific groups will not be tolerated. Furthermore, its inclusion underscores that this violence is condemned because it leads to significant harm to individuals, groups, and communities, simultaneously highlighting that more appropriate and positive community values are required (Hodge, 2011). In short, including S/GBV as a form of hate crime sets a higher bar for the expected behaviors of communities and its citizens who, because of such incidents and legal responses (if any), are given the opportunity to reflect on their own potentially problematic beliefs. As such, the application of hate crime laws, and the often-increased sanctions, emphasize for victims and members of broader society that S/GBV should not be tolerated (Duggan, 2021; Perry & Alvi, 2012). This, in turn, has the potential to increase education and awareness more broadly, which is a second benefit.

#### Increased Public Awareness and Education about S/GBV

Emphasizing S/GBV as a form of hate can provide greater opportunities for increasing public and professional awareness and education (Hodge, 2011). Specifically, it can provide opportunities for deeper discussions about the root causes of S/GBV, its seriousness and prevalence, and how it is exacerbated for some groups of women and girls. This would broaden understandings of S/GBV from a private, individual problem to a broader societal issue with contributors existing at multiple levels, emphasizing the importance of a public health approach to violence prevention (e.g., Heise, 1998). In contrast, omitting S/GBV from hate crime frameworks may reinforce the myth that these forms of violence are less serious than hate-motivated harms against other groups whose victimizations are portrayed as more public, political, and, therefore, more severe (Angelari, 1994).

Increased attention to education and awareness would also challenge long-standing and well-documented attitudes, beliefs, and stereotypes which have served as significant obstacles, and arguably the key obstacle, to the effective prevention of S/GBV (Flood & Pease, 2009). These attitudes, beliefs, and stereotypes are held by individuals and, perhaps more concerning, are entrenched in our institutions, social structures, and social processes. Underscoring when, and how often, S/GBV is motivated by hate can expose these attitudes and more clearly identify far-reaching individual and systemic misogynist attitudes that facilitate and maintain historical and current levels of MVAWG (Gill & Mason-Bish, 2013). For example, while experts recognize that sexual assault is motivated by issues of power and control, the public and the courts continue to largely see these acts as resulting from sexual desire or a need for sexual gratification (Hodge, 2011). Similarly, when a man abuses or kills his female partner, the public, the media, and the courts continue to understand and portray this violence as “a problem in the relationship,” “love gone wrong,” or a “crime of passion” (Dawson, 2006; Fairbairn & Dawson, 2013; Hodge, 2011), making the root causes invisible and precluding the possibility that it may often be violence motivated by hate.

Emphasizing S/GBV as a form of hate, where appropriate, would allow for a deeper consideration of its core contributors which exist at multiple levels. It would further underscore that S/GBV is equally, if not more, supported by societal structures and processes with entrenched misogyny than it is by individual actions. Moreover, an emphasis on the historical and contemporary normalization of many forms of S/GBV, particularly against women and girls, would also underscore the legal severity that surrounds this violence (Brown, 2004), often minimized, especially if it occurs between intimate partners (Dawson, 2006; Hessick, 2007). Finally, such legislation highlights the discriminatory and symbolic aspects of this violence as a social reality for specific groups of primarily female victims as well as the real impacts of S/GBV for the communities to which they belong.

#### Emphasizing Individual and Community Impacts of S/GBV

Hate crime legislation focuses on the impacts of such acts for the individuals targeted and the communities to which they belong, whether it be a racial, religious, or sexual group. As such, treating S/GBV as having the potential to be hate motivated would show the breadth and scope of these negative impacts and consequences for individual victims as well as the community of victims more broadly (Duggan, 2021). For example, individual incidents of S/GBV, such as sexual assault or intimate partner violence, have well-documented negative and damaging impacts on other women in the community, on children and families, and on broader communities and society (Johnson & Dawson, 2011). Each act of S/GBV underscores every woman’s particular vulnerability to such violence, mostly at the hands of men, in public and in private. This is due, in part, to knowledge that much of this violence has its origins in discriminatory and ideological attitudes that devalue, and often support hatred of, women as members of a particular group (Brown, 2004, p. 608). Such impacts are not broadly recognized and could be emphasized using the hate crime framework.

### Challenges

Some of the arguments against including, or emphasizing, S/GBV as hate, or at least those that suggest caution, are overlapping, but each bear separate consideration as they highlight specific elements that challenge the benefits identified above. These challenges are not without their own critiques, however, as will be highlighted.

#### Symbolism is Weak Without Enforcement

Focusing on the symbolic impact, without enforcement, it is argued that the power and impact of including or emphasizing sex, gender, or S/GBV as hate will be weak to non-existent. More specifically, if prosecuting such crimes as hate are to send a message, then failing to prosecute, which often occurs, also sends a message that society does not consider such acts or behaviors as worthy of a response (Gerstenfeld, 2004). In addition, without accompanying education, training, and resources to support real action, this simply provides politicians with the opportunity to stand against such acts with little investment of resources. Put simply, “hate crime legislation is good political capital for little expense” (Hodge, 2011, p. 104). As Jacobs and Potter (1998) highlighted in the United States, hate crime laws in general may represent “more of a political desire to keep the general population happy as opposed to having any real impact on harm education or crime prevention” (pp. 67–68.). However, enforcement is weak for all hate-motivated crimes and prosecution figures are low (Walters et al., 2017). Therefore, one can ask, was this a consideration for other hate motivations preventing them from being identified as such and, if not, why is it important to do so for sex/gender-motivated hate?

#### Criminal Legal Systems will be Overburdened

The sheer number of victims, and it is primarily female victims identified in this argument, has been raised as an objection to including sex or gender as hate motivations (Berard, 2005; Jenness & Grattet, 2005). Often referred to as the “floodgates’or ‘overflow’ argument,” proponents draw attention to several ways that including, or emphasizing, S/GBV as a form of hate—typically discussed with reference to sex or gender—would overwhelm the criminal legal system (Hagerlid, 2021). First, even if not all S/GBV was included, the complexity of identifying which cases were hate motivated would still place significant pressures on legal actors in terms of time and resources (Berard, 2005; Jacobs & Potter, 1998). Second, it is argued that opening hate crime legislation up to sex, or more vigorously pursuing S/GBV as hate-motivated where appropriate, could open the door to demands for provisions based on other identities, further overloading the system (Jenness & Grattet, 2005).

There are several critiques of these arguments. First, one might argue that the expectation of a “floodgate” of cases involving S/GBV as hate is proof in and of itself that S/GBV should be a focus for hate crime legislation or, where it is already possible, more often investigated and prosecuted as a hate than is currently the case. Relatedly, if one assumes this is correct, it must be asked, when has a crime, particularly a violent crime, not been considered worthy of an appropriate response by virtue of its sheer volume of incidents? Arguably, some forms of S/GBV such as domestic violence and various types of sexual assault/harassment are good examples of where this has occurred, given these crimes experience such low response rates (Johnson & Dawson, 2011). However, in general, if an act or behavior is considered a crime, or a hate crime, it should be treated as such, regardless of how often it occurs.

Second, challenging the assumption that there would be a slew of cases overwhelming the system, where it is possible to pursue sex-motivated violence as hate, this has not occurred. In fact, the opposite is true, which is a problem in and of itself. In the United States, for example, some prosecutors did not even know that gender was a protected characteristic (Hodge, 2011: McPhail & DiNitto, 2005), underscoring the need for increased awareness, education, and training. Third, this argument does not recognize that to include, or emphasize, S/GBV as potentially hate motivated does not mean that all S/GBV will be treated as such. Like other hate-motivated acts of violence, there would be a limited set of cases whereby sex/gender-motivated hatred can be clearly shown as the driving motivation or part of the motivation. This would require that the perpetrators clearly expressed hatred and contempt for women in some way, which might include evidence of high levels of brutality, fatal violence, acts against a series of victims, and/or other criteria that was deemed to denote hate (Berard, 2005; Duggan, 2021). These criteria are returned to below.

Fourth, there is no evidence that it would be more difficult to establish S/GBV as a form of hate than the burden of proof required for other hate crimes such as those motivated by race or religion. The lower prosecution rates for all hate crimes suggests that possibility, although it could also suggest the lack of recognition of hate crimes overall. Finally, with respect to opening the door to many other factors, this is already possible in Canada where “other similar factors” is included in legislation. As Perry (2016) argues, Canada is seemingly unique by including this reference which allows for hate targets who have been historically discriminated against as well as “emerging” identities not currently named in the legislation (p. 593). However, there is no evidence that “other similar factors” have invited numerous other groups to argue for hate motivations.

#### Resource Intensiveness of Emphasizing S/GBV as a Form of Hate

Similar to the “floodgates argument, is highlighting that the extent of resources required to address S/GBV as hate would be significant given the sheer number of potential cases that may need to be considered (Gerstenfeld, 2004; Hodge, 2011). When discussing any criminal justice initiative, or prevention initiatives more generally, the need for adequate resources is typically part of the discussions. This is no less true when considering the inclusion of, or emphasis on, S/GBV as hate. However, resources should not determine what is responded to as a crime, or treated as a hate crime if it is clearly motivated by hate. Furthermore, if significant resources are required, this may stem from the lack of clarity in the legislation and/or vague criteria to determine what is to be considered S/GBV as a form of hate. With clear laws and criteria, not all S/GBV would be categorized as such, reducing the extent of resources required with a significant proportion of cases continuing to be handled through current mechanisms.

#### Challenge of Training Legal Actors

One aspect of the time and resources required is related to the challenge of training legal actors to understand, and be capable of identifying, S/GBV as a form of hate when it occurs. This challenge is exacerbated by entrenched and well-documented sexism that continues to reign in the still largely patriarchal, legal system (Angelari, 1994; Duggan, 2021). For example, after decades of work, legal professionals still often fail to understand the power and control aspects of S/GBV (e.g., coercive-controlling tactics), so what is the likelihood that they can be trained to recognize hate-motivated S/GBV. Similarly, despite feminists and others’ best efforts, victim-blaming attitudes continue to dominant even after years of social and legal transformations (van der Bruggen & Grubb, 2015). There would have to be a greater emphasis on the extent, and quality, of training as a result. For example, as noted by Perry (2016), in Canada and the United States, of the 12 weeks to 6 months of training received by new police recruits, less than 20 hours focuses on diversity training which could cover a variety of topics, but little to no time is dedicated to hate crimes (p. 596), so it is unlikely that much time is currently spent on acts of hate motivated by sex.

#### S/GBV is Already Addressed by Existing Legislation

It is argued that existing laws already address the disproportionate impact of S/GBV on women, such as non-specific offences that capture sexual assault and domestic violence (e.g., common assault, assault causing bodily harm). The ability to add S/GBV offenses outside the hate crime framework falls within this argument as illustrated by the U.K. Law Commission (2021) who recently concluded that this was a better option than including sex or gender. “Instead, more target options outside the hate crime framework—such as a possible offence of public sexual harassment—should be considered to address some of the specific concerns that have driven calls for misogyny to be included with hate crime laws” (p. 127). Notwithstanding the fact that existing laws have been deemed largely ineffective by research too voluminous to include here, research globally has documented that much S/GBV is not treated as serious within the legal system, even when it ends in death, especially if men kill their female partners (the latter referred to as the “domestic discount or ‘intimacy discount’; Dawson, 2016; Rapaport, 1994). Moreover, for most other hate crime offences, the underlying offence is also already punishable by criminal law; therefore, if this fact does not invalidate recognition of other hate motivations, why would it invalidate a sex-motivated hate that leads to violence? Finally, existing laws often fail to highlight discriminatory aspects of these crimes which may be done more effectively through hate crime frameworks by underscoring how such acts are produced by ongoing sex/gender inequalities experienced by women as a group.

#### S/GBV is Different From Other Hate Crimes

The argument that S/GBV is different from other hate crimes is a common one and multi-faceted. For example, when the U.K. Law Commission (2021) recommended that sex and gender not be included in hate crime laws, they stated “crimes connected with sex and gender characteristics raise unique issues that are not present to the same extent in relation to the existing five characteristics protected in hate crime laws” (p. 127). Various elements of this argument apply to sex/gender-motivated hate against women and girls as discussed below.

*Women are not a minority*. It is argued that women do not constitute a minority and, as such, should not be included in hate crime legislation. However, it could be argued that some women, depending on overlapping identities, do comprise a minority (e.g., Indigenous or Black women in some countries) and are at an increased risk of hate-motivated S/GBV. This argument also assumes that those most often represented in hate crime statistics continue to represent a minority which, with changing demographics in various jurisdictions, may no longer be the case. Finally, women may not be a “minority” in numbers, but they are a minority in power given historical and ongoing oppressions and sexism in a patriarchal society and in law.

*Women and the “intimacy problem”*. Hate crimes are most often understood as involving strangers (Mason, 2014; Stanko, 2001), whereas S/GBV is most often perpetrated by those known to the victims (e.g., male partners or family members). Referred to as the “intimacy problem” (Gelber, 2000), there are several critiques of this argument. First, there is the assumption that these types of cases more often involve interpersonal conflict rather than hate or misogyny. However, it is recognized that S/GBV, and male-perpetrated violence against women specifically, has deep historical, cultural, and legal roots that can be categorized as hate or misogyny (Sarmiento et al., 2014). Is it any more difficult to view a man who rapes and murders his wife as a hate crime than the rape and murder of a woman by a male stranger as she walks home from work? If the answer to that question is yes, we must deconstruct and challenge why that is the case.

Second, this argument begs the question of why hate crimes are more often perceived to involve strangers and whether, in fact, they do (Burman et al., 2009). For example, Walters and Hoyle (2011) have argued that this stereotype of hate crimes leaves out “the messier and sometimes intractable disputes between neighbors, colleagues, and other acquaintances . . .” (p. 8). More research is needed to examine this contention which is expected to challenge the notion of hate crime as typically stranger-perpetrated acts. Third, a significant proportion of S/GBV does not involve a known perpetrator and even those cases are rarely seen as S/GBV as a form of hate. The examples described at the beginning of this article are clear evidence of this fact. Finally, more than two decades ago, Gelber (2000) argued that “the inclusion of the stranger factor in some hate crime definitions demonstrates the singular success of the normalization of much violence against women.” That is, the intimacy problem was created by definitions of hate crime and should not override or negate women’s shared experiences of hate (see also Haynes & Schweppe, 2020; Mason-Bish, 2013).

*Women are not interchangeable*. A key impact of hate crimes is that other members of the community live in fear that it could happen to them; however, because women and girls often have prior relationships with their perpetrators, it is argued that women and girls are not “interchangeable” because “not any woman (or girl) would do.” As noted above, women do fear for their own safety when another woman in the community is the victim of violence, whether the attacker is known to the woman or not. Moreover, not all female victims of violence have prior relationships with their attackers and not all perpetrators restrict their violence to specific and individual women.

*S/GBV is motivated by power and control*. Feminists have worked hard to increase education and awareness about the way in which S/GBV, particularly in women’s relationships with men, is motivated by a desire for power and control (Pence & Paymar, 1993). An emphasis on the motivation of power and control represents progress from a time when such violence was most often seen as a private “domestic problem” or attributed to mental illness of male perpetrators. However, we need to ask whether power and control and hate are mutually exclusive? Could it not be argued that a man’s desire to impose power and control over a female partner is, in part, an expression of hate? This is an important question given that, in one of the few studies examining this issue, McPhail and DiNitto (2005) found that prosecutors saw power and control (and, sometimes, love), rather than hate, as the chief motivations for S/GBV. They wrote:

“. . . the superficial adoption of the term *power and control* by prosecutors demonstrates a lack of a deeper understanding of VAW. For fueling VAW is misogyny for women as a group, and this includes discriminatorily selecting females for attack, a hallmark of hate crime. Viewing VAW as *only* because of motivations of power and control leads to a failure to see multiple causations of VAW and precludes the adoption of the perspective that VAW is a hate crime. Adopting a hate crime perspective does not negate including motivations of power and control. But using only the power and control lens limits a fuller understanding of VAW and girls and innovative approaches to addressing it” (p. 1,180).

#### Emphasis on Penalties Rather Than Alternative Approaches

Some research has found that those who are against, or at least cautious about, including or emphasizing S/GBV as a form of hate, including some prosecutors and feminist advocates, are concerned about increasing punitive impacts on perpetrators (Hodge, 2011; McPhail & DiNitto, 2005). It is argued that increased sanctions do not deter crime or offenders, and such sanctions are typically imposed on perpetrators from already over-criminalized populations. To counter the emphasis on penalties, alternative approaches have been emphasized in some literature (e.g., restorative justice approaches, civil remedies). For example, in a 3-year examination in the United Kingdom, Gavriedlides (2012) found that restorative justice might offer one type of mechanism that would help “break down the fears, stereotypes, and causes of hate crime,” particularly for minor acts which could lead to serious future acts without early intervention (p. 3,640). This concern aligns with increasing calls in many countries to defund police alongside a growing prison abolition movement (e.g., see Chartrand, 2021; Pasternak et al., 2022; Wortley & Owusu-Bempah, 2022). Thus, what is needed is *better* responses to hate crime, including those that emphasize public awareness and education, rather than *more* laws and *more* criminalization. These arguments apply to all forms of hate that lead to penalty enhancements, however, and not just those that would accrue if there was a greater emphasis on S/GBV as a form of hate.

## Discussion and Conclusion

During the past decade, there have been countless incidents of lethal and non-lethal violence perpetrated against women with demonstrated misogynistic motives, grounded in, and facilitated by broader sexist, patriarchal, and misogynist ideologies, social structures, and processes. Seldom have these incidents been discussed as, or attributed to, S/GBV as hate, or considered hate crime, by those working in the legal system, by politicians, by the media, or by broader publics. Given this, it is expected that the bulk of S/GBV motivated by hate perpetrated against women and girls because of their sex or gender, both online and offline, has largely remained under the radar. This raises the question asked at the beginning of this article, why S/GBV as a form of hate rarely, if ever, qualifies as hate crime even when the related characteristics of sex or gender are included in legislation, and significant commonalities exist with more recognized targets of hate crime. The answer to this question is crucial since acts of hate impact one’s health and well-being as well as participation in both private and public life, an issue more pressing with the rise of online hate against women and girls.

This article argues that, to date, it has been purely symbolic when sex or gender is included as protected characteristics or identifiable groups given how few crimes are recorded and prosecuted with this motivation. As such, it is important to underscore that “the extent to which law can provide an effective impetus for social change varies according to the conditions present in a particular situation” (Vago and Nelson, 2013, p. 312). According to Vago and Nelson (2013), some of these conditions are how much information is known about the legislation, whether there is a clear and concise statement in the legislation that precludes varying interpretations, and how responsive are those tasked with enforcing and implementing the law? While there are valid arguments for and against the inclusion of, or emphasis on, S/GBV as a form of hate, and the related characteristics of sex or gender, what is largely absent is systematic, empirically based evidence examining many of the issues discussed (Hagerlid, 2021). As a result, drawing from Vago and Nelson (2013), and recognizing the lack of knowledge about, or consideration of, S/GBV as a form of hate globally, this article identifies four broad research and policy priorities which can further (or arguably begin) the conversation about the role of hate in S/GBV motivated by sex or gender.

### Enhanced Monitoring of Hate Motivated by Sex, Gender, Including S/GBV

Governments and data collection agencies need to enhance monitoring of S/GBV as a form of hate, including reporting, recording, prosecuting, and sentencing. This would entail using accurate terminology when discussing S/GBV as a form of hate, including whether groups were targeted because of their sex/gender, sexual orientation, and/or gender identity or expression. This is crucial to determine similarities and differences which will underpin nuanced and effective interventions and preventions. Such monitoring would also require the ability to account for the complexities of hate experiences, from the perspective of the victims, which often involve multidimensional motivations and intersectional experiences of those targeted. Efforts at enhanced monitoring could begin with a focus on specific forms of S/GBV as hate crime such as rape/sexual assault or femicide and/or a dedicated focus on one or two jurisdictions to pilot more nuanced and targeted data collection instruments to better capture experiences of hate-motivated violence.

### Examining Current Understanding of Sex, Gender, and S/GBV as Hate

Given its relative invisibility as a hate crime, it is important to examine what are current understandings of S/GBV as a form of hate. This would include documenting legal actors’ understandings and perceptions of hate crime, including the role of sex or gender, and S/GBV. As noted above, prosecutors in a U.S.-based study were not even aware that gender was a protected characteristic. It is also recommended that the concerns of those who work with women such as anti-violence against women organizations as well as victims of S/GBV themselves are examined, including these victims’ own understandings of their experiences as a form of hate. While all of the above groups are members of the public as well, broader examinations of attitudes, beliefs, and stereotypes held by the general public are also key to understanding whether and how S/GBV can be considered a form of hate and how sex or gender are related. To adequately consider sex, gender, and S/GBV as targets of hate or hate motivated, victim knowledge is key to addressing underreporting and knowledge of legal actors is crucial to adequately responding when reports are made. In addition to the role of primary prevention to achieve such goals (i.e., increasing awareness and education), identifying what mechanisms are currently in place and how they might be improved is also vital as discussed next.

### Building on Existing Mechanisms Responding to Sex, Gender, and S/GBV as Hate

There are some existing mechanisms that are capable of responding to S/GBV as a form of hate. For example, misogyny has been considered as a potential motivation for hate crime by some jurisdictions (Zempi & Smith, 2022). Proponents of this approach argue that prioritizing misogyny as a categorization, rather than sex or gender, recognizes the specific risks and vulnerabilities faced by women and girls due to individual and structural patriarchal oppressions (Gill & Mason-Bish, 2013; Mason-Bish & Duggan, 2019). The idea of including misogyny as a hate crime is not new but with recent incel-motivated killings of primarily women, it has received more attention globally, largely in jurisdictions where sex or gender is not currently included in legislation. This possibility was rejected in the United Kingdom, but it is still under consideration in Scotland. Others argue that it would be constitutionally inappropriate to include misogyny in hate crime legislation to the exclusion of other gendered forms of hate and argue the inclusion of more neutral characteristics such as sex or gender is more constitutionally sound (Haynes & Schweppe, 2020, pp. 289–290).

From a practical perspective, there is no consistent or agreed upon definition of misogyny, so it may be difficult to address as a hate crime (Duggan, 2021; Mullany & Trickett, 2018). Globally, however, there has been a call for misogyny to be seen as a possible “gateway” to terrorism and to pay more attention to misogyny and its interrelationship to all other forms of terrorism (United Nations Development Programme, 2021). In the Canadian context, for example, misogyny has recently been embedded as a form of violent extremism with the addition of misogynist groups to listings of terrorist entities (FINTRAC Special Bulletin, 2021). It might make sense, then, to also consider misogyny as type of ideological hate that disproportionately impacts women and girls, and forms of S/GBV perpetrated against them.

S/GBV as a form of hate may also be included as specific crimes or in specific contexts. These might include femicide; sexual violence, including rape or sexual assault; sex trafficking; prostitution/sex work; forced marriage; female genital mutilation; domestic/intimate partner violence; criminal harassment; strangulation; and coercive controlling behaviors. Latin America, for example, has led the way in focusing on femicide offenses, in some cases as a form of hate. Some countries have classified a death as femicide when it happened “as a result of her condition as a woman,” which is the case of Colombia (Law 1257 of Colombia), or when “motives of hate or contempt for her as a female” are factors as is the case in El Salvador (Decree No. 520 of 2010 of El Salvador). Mexican and Honduran legislation have established that a crime rises to femicide when the death was produced for “gender-related reasons” (Decree of June 13, 2012, of Mexico; Decree No. 23-2013 of Honduras). Finally, in Argentina, femicide includes hate motivated by gender or sexual orientation and, in Guatemala and Nicaragua, femicide offences include those motivated by misogyny (Sarmiento et al., 2014, p. 54).

Finally, in various jurisdictions, courts can apply hate as an aggravating factor at sentencing, although this rarely occurs for hate motivated by sex or gender. For example, the Department of Justice Canada examined three decades of case law and found that sex was relatively invisible. The authors acknowledged published case law does not represent all adjudicated cases, but more often those that are precedent-setting or are related to more serious crimes. However, it is also likely that because sex so seldom appears in case law as a victimization ground, it would be more likely to end up in reported case law as precedent-setting. In countries where this option exists, it provides a potential mechanism to recognize and highlight S/GBV as a form of hate, underscoring the societal condemnation of such acts for victims of specific crimes, but also of the entrenched hostile and prejudicial attitudes that support such violence (Maher et al., 2015).

The above existing legislation and related policies and practices require improvements, at the very least, and more systematic, evidence-based research examining their use and effectiveness. This would, in the first instance, require understanding whether there are clear criteria for identifying when it is appropriate to respond to S/GBV as a form of hate, including the role of sex and gender. In the case of femicide, for example, a recently released international statistical framework identifies 10 factors indicative of femicide, one of which is a gender-based hate crime in which the victim “was targeted because of a specific bias against women on the part of the perpetrator(s)” (UNODC, 2018, p. 12). The framework argues that “signs of hate crime can be recognizable by the specific modus operandi or context” including:

“an attack on a woman who was previously engaged in activism in support of women’s rights; an attack on a woman by a perpetrator who used insults and offensive words towards her for being a woman; an attack on a female group/organization; an attack (or series of attacks or killings) that primarily targets women; an attack on a (LGBTI) woman by a perpetrator who used insults and offensive words towards her sexual orientation or gender identity, in written format or in other ways; an attack on a woman by a perpetrator who had used messages of hatred against women, in written format or in other ways; an attack on a woman by someone known to her (such as a colleague or neighbor), in which she is the direct victim of the perpetrator’s animosity, which is underpinned by wider misogyny; an attack on a woman by a perpetrator who belongs to a hate group that specifically targets women” (pp. 13–14).

The above is a starting point only because it arguably still largely renders invisible sex-motivated hate that leads to male violence against female partners, but it does begin to identify in more concrete terms what is meant by a “gender-based hate crime.” This also addresses a common critique of femicide laws, as stated by the *Inter-American Model Law on the Prevention, Punishment and Eradication of the Gender-Related Killing of Women and Girls (Femicide/Feminicide)* (Organization of American States, 2018):

Since determining the subjective factors that comprise the intent of the attacker poses a complicated challenge for agents of the criminal justice system in terms of standards of proof, the Committee has undertaken to establish the objective acts that should be considered to determine the existence of gender-based motives, from an intersectional perspective, as well as the social context, the community of the victim and the perpetrator, including their cultural and religious beliefs, and thereby eliminate the need to determine the state of mind or any individual motive (*mens rea*) on the part of the attacker (p. 19).

It is not clear whether other forms of more well-recognized hate crimes such as those targeted because of their race or religion have such criteria, but such a move for all hate crime would ensure more consistent application of laws moving forward. Along with clear criteria, consistent and quality training for legal actors is also necessary to improve responses as is the development of public awareness and education campaigns to help to promote the reality that S/GBV can be hate motivated.

In summary, this article has underscored the absolute and relative invisibility of S/GBV as a form of hate in both law and in society globally, particularly for women and girls. The public and professionals, including academia, appear to be almost completely unaware of the extent of such hate-motivated violence. The current treatment of S/GBV—including sex or gender as motivations for hate—as not deserving the same recognition as other forms of hate-motivated violence speaks volumes, not only to the millions of women and girls who are victims of S/GBV but also to the millions who fear daily such victimization. It is recognized that legislation which targets problematic behaviors can only do so much and that real change needs to come at structural and practical levels. Legislative frameworks that recognize and respond to S/GBV as a form of hate, including sex or gender, is the first step in social change at the structural and then, hopefully, practical level. Recognizing sex-motivated hate that leads to violence can help shift the individual and cultural focus from the behavior of the victim (e.g., victim-blaming) to the behavior of the perpetrators, including how individual misogyny as a motivation is often underpinned by systemic and institutionalized misogyny. Until then, S/GBV as a form of hate remains marginalized in hate crime legislation—where such cases are notably rare, especially related to sex or gender, if included at all—just as women and girls remain invisible as victims of violence in society more generally.
